# Dietary ferulic acid supplementation enhances antioxidant capacity and alleviates hepatocyte pyroptosis in diquat challenged piglets

**DOI:** 10.1186/s40104-024-01086-5

**Published:** 2024-10-07

**Authors:** Junqiu Luo, Xiu Wu, Daiwen Chen, Bing Yu, Jun He

**Affiliations:** Institute of Animal Nutrition, Key Laboratory for Animal Disease-Resistance Nutrition of China Ministry of Education, Sichuan Agricultural University, Chengdu, Sichuan 611130 People’s Republic of China

**Keywords:** Antioxidant capacity, Ferulic acid, Hepatic pyroptosis, Piglets

## Abstract

**Background:**

Oxidative stress significantly impacts growth performance and liver function in piglets. Ferulic acid (FA) works as an antioxidant, however, the role and mechanism of FA in the regulation of diquat-induced oxidative stress in piglets are less known. This study was designed to investigate the effects of FA on growth performance and antioxidant capacity in piglets with diquat challenge.

**Methods:**

Thirty-two healthy DLY (Duroc × Landrace × Yorkshire) piglets (13.24 ± 0.19 kg) were randomly divided into one of two diets including 0 or 4 g/kg FA for 14 d. On d 15, all pigs were intraperitoneally injected diquat or sterile saline.

**Results:**

Dietary supplementation with ferulic acid (FA) significantly improved the average daily gain (ADG) and decreased feed-gain ratio (F/G) of piglets. Here, dietary FA supplementation reduced serum aspartate aminotransferase (AST), alanine aminotransferase (ALT) activities in diquat challenged piglets. Furthermore, diquat infusion increased reactive oxygen radicals (ROS) level in liver, decreased the activities of total superoxide dismutase (T-SOD) and glutathione peroxidase (GSH-Px), total antioxidant capacity (T-AOC) and increased malondialdehyde (MDA) content in the liver and serum. Supplementation with FA significantly increased T-AOC and T-SOD activities and decreased MDA and ROS levels. FA down-regulated gene and protein expression of Keap1, and up-regulated protein expression of Nrf2 and HO-1 in the liver of piglets with diquat challenge. Importantly, diquat challenge increased the ratio of late apoptosis, increased serum levels of IL-1β, IL-18 and lactate dehydrogenase (LDH), and up-regulated pyroptosis-related genes in the liver. FA supplementation reduced the ratio of late apoptosis and down-regulated mRNA expression of *Caspase-1*. Accordingly, FA addition reduced concentration of IL-1β, IL-18, and LDH under diquat challenge.

**Conclusions:**

Diquat-induced oxidative stress reduced growth performance and impaired liver function in piglets. Dietary FA supplementation enhanced the antioxidant capacity and reduced the degree of hepatocyte pyroptosis, thereby alleviating the oxidative damage in the liver and mitigating the impact of diquat on growth performance of piglets.

## Introduction

Oxidative stress is a physiological state in which an organism, when exposed to various external stimuli or endogenous factors, generates an excessive amount of reactive oxygen radicals (ROS), resulting in a disruption of cellular redox homeostasis. In vivo elevated ROS is typically accompanied by increased levels of inflammatory cytokines, which further promote apoptosis and pyroptosis [1]. As an essential metabolic organ, liver exhibits high susceptibility to oxidative stimuli due to its intricate biochemical character [1, 2]. Oxidative stress-induced liver injury not only leads to inferior disease resistance and poor health status, but also affects growth and development in piglets [3–5]. Therefore, understanding of the impact of oxidative stress on piglets and finding effective antioxidant agents are critical.

Ferulic acid (FA, 4-hydroxy-3-methoxycinnamic acid) exhibits diverse biological activities, including antioxidant, anti-inflammatory, anti-tumor and antibacterial effects [6–9]. FA mitigates oxidative stress by activating antioxidant enzymes and scavenging free radicals [10]. FA facilitates nuclear translocation of Nrf2, activates the Nrf2 signaling pathway and up-regulates the transcription and expression of the HO-1. This mechanism attenuates the oxidative damage induced by radiation in the liver and lymphocytes of mice [11, 12]. FA has also been observed to prevent methotrexate-induced hepatotoxicity and alleviate oxidative stress, inflammation and apoptosis by enhancing Nrf2/HO-1 signaling in rats [13]. In recent study, FA has been found to reduce TNF-α-induced damage in human intestinal microvascular endothelial cells and prevent inflammatory injury in endothelial cells of mice with ulcerative colitis by inhibiting the TXNIP/NLRP3 signaling pathway [14]. However, limited information is available on the effects of FA supplementation in piglets with hepatic injury induced by oxidative stress.

Therefore, our study aimed to confirm whether FA supplementation can improve antioxidant status and alleviate inflammation in piglets under diquat challenge. The results provide guidance for the application of FA in the prevention and treatment of oxidative damage in swine industry.

## Materials and methods

All animal experiments were approved by the Institutional Animal Care and Use Committee of the Laboratory Animal Center at Sichuan Agricultural University (No. 20220815).

### Materials

Ferulic acid (purity ≥ 99%) and diquat (purity ≥ 99%) were purchased from Shanghai Aladdin Biochemical Technology Co., Ltd. (Shanghai, China). A sterile physiological saline solution containing 10 mg/mL of diquat was prepared and available for use.

### Animals, diet and experimental design

Thirty-two piglets (Duroc × Landrace × Yorkshire) with an average body weight of 13.24 ± 0.19 kg was selected and randomly divided into 2 groups with 16 replicates, which were respectively fed basal diet (CON group) and the basal diet containing 4 g ferulic acid per kg of feed (FA group). The basal diet (Table 1) was formulated in accordance with the nutrient requirements specified by the National Research Council (2012) [15]. On d 15, piglets within each group were further randomly divided into 2 sub-groups with intraperitoneal injection of sterile saline and diquat respectively. Thus, from 15 d, pigs were randomly allotted into four treatments with 8 pens per treatment and 1 pig per pen. The 4 treatment groups include control, FA, diquat, and (FA + diquat) group. The CON and FA groups received intraperitoneal injection of sterile saline (0.9%), whereas the diquat and (FA + diquat) groups received injection of diquat (10 mg/kg BW). Pigs were individually housed in stainless-steel crates (1.8 m × 0.8 m × 1.6 m; temperature 26–28 °C; humidity of 45%–65%) and fed at 8:00, 14:00 and 20:00 ad libitum with free access to water. On d 20, after weighing and blood collection, all pigs were euthanized for sampling.

**Table 1:** Composition and nutrient levels of basal diet, %

Ingredients	Percentage	Nutrient level^3^	Content
Corn (CP 7.8%)	62.34	DE, Mcal/kg	3.31
Wheat bran	4.50	CP, %	17.75
Soybean meal (CP 46%)	19.80	Calcium, %	0.70
Extruded Soybean (CP 35.5%)	5.00	Total phosphorus, %	0.55
Fish meal (CP 62.5%)	2.00	Available phosphorus, %	0.33
Soybean oil	1.50	Lysine, %	1.10
Dicalcium phosphate	0.78	Methionine, %	0.46
Limestone	0.69	Methionine + Cysteine, %	0.68
NaCl	0.30	Threonine, %	0.73
Sucrose	2.00	Tryptophan, %	0.20
Methionine (99%)	0.19		
L-Lysine·HCl (98%)	0.30		
Threonine (98.5%)	0.14		
Tryptophan (98%)	0.01		
Choline (50%)	0.10		
Vitamin premix^1^	0.05		
Mineral premix^2^	0.30		
Total	100.00		

*CP* Crude Protein, *DE* Digestible energy

^1^The vitamin premix provided the following per kg of diet: 9,000 IU of VA, 3,000 IU of VD_3_, 20 IU of VE, 3 mg of VK_3_, 3 mg of VB_1_, 4 mg of VB_2_, 3 mg of VB_6_, 0.2 mg of VB_12_, 30 mg of niacin, 15 mg of pantothenic acid, 0.75 mg of folic acid, and 0.1 mg of biotin

^2^The mineral premix provided the following per kg of diet: Fe (FeSO_4_·H_2_O), 100 mg; Cu (CuSO_4_·5H_2_O), 6 mg; Mn (MnSO_4_·H_2_O), 4 mg; Zn (ZnSO_4_·H_2_O), 100 mg; I (KI), 0.14 mg; Se (Na_2_SeO_3_), 0.3 mg

^3^Nutrient level values were calculated

### Growth performance parameters

The individual feed intake was recorded every day, and the individual body weight (BW) was measured on d 1, 15 and 20. The average daily gain (ADG), average daily feed intake (ADFI) and feed:gain ratio (F/G) were calculated for the 1–14 and 15–19 days of the experiment.

### Sample collection

Blood samples were collected from the anterior vena cava of each piglet. Then, serum samples were centrifuged at 3,000 × *g* for 15 min at 4 °C and stored at −20 °C for further analysis. Following blood collection, the piglets were euthanized and promptly slaughtered. The middle part of the left lobe liver tissue (5 mm × 5 mm × 5 mm) was collected from each pig and preserved in 4% paraformaldehyde for morphological analysis. Additionally, another portion of liver tissue (2 g) was placed in a sterile tube, rapidly frozen in liquid nitrogen, and then stored at −80 °C for further analysis.

### Liver morphology examination

The liver tissues were placed in 4% formaldehyde buffer for 24 h, transferred to 70% ethanol solution, embedded in paraffin, and subsequently stained with hematoxylin and eosin (H&E). After H&E staining, pathological changes were performed using a light microscope (Nikon ECLIPSE 80i; Nikon Corporation, Tokyo, Japan).

### Serum biochemical analysis and inflammatory cytokine detection

Serum aspartate aminotransferase (AST), alanine aminotransferase (ALT) and lactate dehydrogenase (LDH) were measured using commercial kits (Nanjing Jiancheng Bioengineering Institute, Nanjing, China). The levels of interleukin-1β (IL-1β) and interleukin-18 (IL-18) in serum were assayed with ELISA kit (Jiangsu Meimian Industrial Co., Ltd., Yancheng, China). The methods were followed by the manufacturer’s instructions.

### Measurement of the antioxidant parameters

Serum and liver total superoxide dismutase (T-SOD) and glutathione peroxidase (GSH-Px) activities, total antioxidant capacity (T-AOC) and malondialdehyde (MDA) concentrations were determined using assay kits (Nanjing Jiancheng Bioengineering Institute, Nanjing, China). Total protein content was determined with bicinchoninic acid (BCA) protein assay kit (Beyotime Biotechnology, Shanghai, China). The methods were measured in accordance with the manufacturer’s instructions.

### Flow cytometry assays

Reactive oxygen species (ROS) and apoptosis were measured by flow cytometry. Fresh liver samples were crushed and centrifuged at 600 × *g* for 5 min at 4 °C. The resultant pellet was treated with trypsin (DMEM + Collagenase A + DNase) and thoroughly shaken for 60 min. After complete digestion, the cell suspension was filtered through 70 μm sieve. Subsequently, the cell concentration was adjusted to 1 × 10^6^ cells/mL using phosphate-buffered saline (PBS).

Then, 100 μL of cell suspension was incubated with 5 μmol/L DCFH-DA (ROS fluorescent probe, Solarbio, Beijing, China, 4091-99-0) working solution for 30 min at 37 °C. The cells were washed with PBS and centrifuged at 600 × *g* for 5 min. Finally, the cell precipitate was resuspended with 500 μL PBS. The fluorescence intensity was detected by flow cytometry (FACSVerse, BD Biosciences, NJ, USA).

Apoptosis was analyzed using the Annexin V-FITC/PI Apoptosis Detection kit (BD Biosciences, NJ, USA, 556547). A total of 100 μL cell suspension was mixed with 5 μL Annexin-V and 5 μL propidium iodide (PI), stained for 15 min at room temperature, and then washed with PBS and centrifuged at 4 °C for 5 min. The resulting cell pellet was resuspended in 500 μL of PBS, and finally detected by flow cytometry.

### Real-time PCR and Western blotting

Total RNA was extracted from liver samples using RNAiso Plus reagent (TaKaRa, Dalian, China). RNA concentration was determined spectrophotometrically. Subsequently, reverse transcription was performed using the PrimeScript^TM^ RT Reagent kit (TaKaRa, Dalian, China). Real-time PCR was measured with the SYBR Green PCR reagent kit (TaKaRa, Dalian, China) using the QuantStudio 5 Flex real-time PCR system (Applied Biosystem, Foster, CA, USA). The primer sequences are shown in Table 2. β-Actin was used as the housekeeping gene. Relative gene expression levels were evaluated using the 2^−∆∆Ct^ method.

**Table 2:** Primer sequences

Genes	Primers	Sequences (5′→3′)	Accession numbers	Product length, bp
*β-actin*	Forward	TGGAACGGTGAAGGTGACAGC	XM_003124280.5	177
	Reverse	GCTTTTGGGAAGGCAGGGACT		
*Keap1*	Forward	TGCACGCTGCGATGGAG	NM_001114671.1	118
	Reverse	GCATGGGGTTCCAGATGACA		
*Nrf2*	Forward	GCCCCTGGAAGCGTTAAAC	XM_021075133.1	67
	Reverse	GGACTGTATCCCCAGAAGGTTGT		
*HO-1*	Forward	TACCGCTCCCGAATGAACAC	NM_001004027.1	140
	Reverse	TGGTCCTTAGTGTCCTGGGT		
*NQO1*	Forward	TGTAAAGCCGGGAAAGGTGT	NM_001159613.1	132
	Reverse	CCATTGAGGAGTTGGGTGCT		
*NLRP3*	Forward	GGAGGAGGAGGAAGAGGAGATA	NM_001256770.2	147
	Reverse	AGGACTGAGAAGATGCCACTAC		
*ASC*	Forward	ACAACAAACCAGCACTGCAC	XM_003124468.5	126
	Reverse	GCTGGCATTTGTGGGAAGAAATACTC		
*Caspase-1*	Forward	GCTGGCATTTGTGGGAAGAAATACTC	NM_214162.1	140
	Reverse	CCACGGCAAGCCTGGATAATGATC		
*GSDMD*	Forward	GCCTGAGCACAAAGTCCTGC	XM_021090504.1	85
	Reverse	CTCCTTCTGCGTCTGGAGCA		
*IL-1β*	Forward	CAGCTGCAAATCTCTCACCA	NM_214055.1	112
	Reverse	TCTTCATCGGCTTCTCCACT		
*IL-18*	Forward	AGTAACCATCTCTGTGCAGTGT	XM_005667327.2	155
	Reverse	TCTTATCATCATGTCCAGGAAC		

*ASC* Apoptosis-associated blotch-like proteins, *Caspase-1* Cysteinyl aspartate specific proteinase-1, *GSDMD* Gasdermin-D, *HO-1* Heme oxygenase 1, *IL-1β* Interleukin-1β, *IL-18* Interleukin-18, *Keap1* Kelch-like ECH-associated protein 1, *NLRP3* Nod-like receptor protein 3, *NQO1* NAD[P]H-quinone oxidoreductase 1, *Nrf2* NF-E2-related factor-2

RIPA Lysis Buffer (Beyotime, Shanghai, China) was used to extract protein from the liver samples, and then the protein content was quantified using the BCA protein assay kit following manufacturer’s instruction. The liver protein samples were separated with 10% SDS polyacrylamide gel electrophoresis (SDS-PAGE) and transferred onto polyvinylidene difluoride (PVDF) membranes (Millipore, Eschborn, MA, Germany). The PVDF membranes were blocked with 5% skim milk powder for 60 min at 25 °C. Then the membranes were incubated overnight at 4 °C with primary antibodies. After rinsing, the membranes were incubated with secondary antibodies for 1 h at 25 °C. Protein signals were visualized using BeyoECL Plus (Beyotime, Shanghai, China) and captured on ChemiDoc XRS imaging system. Image Lab software (Bio-Rad, Berkeley, CA, USA) was utilized for quantification of the immunoblotting results. The specific experimental procedures were conducted in accordance with the methods described by Wang et al. [16]. The antibodies include Keap1 (Proteintech, Chicago, IL, USA, 10503-2-AP), Nrf2 (Proteintech, Chicago, IL, USA, 16396-1-AP), HO-1 (Proteintech, Chicago, IL, USA, 10701-1-AP) and β-actin (Cell Signaling Technology, Beverly, MA, USA, 3700).

### Statistical analysis

Data were analyzed via SPSS 27.0 (Chicago, IL, USA). The growth performance of piglets prior to diquat injection was analyzed by Student’s *t*-test. The growth performance followed by injection was analyzed as a 2 × 2 factorial with the general linear model procedures. The factors of model include the main effects of FA treatment and diquat challenge as well as their interaction. The other data were analyzed by one-way ANOVA test with Duncan’s multiple comparison. All data were tested by means of normal distribution. A value of *P* < 0.05 was considered statistical significance.

## Results

### Growth performance

The effects of FA supplementation on growth performance of piglets are presented in Table 3. During a 14-d period of FA feeding, piglets exhibited a significant increase in ADG and a decrease in F/G compared with the control group (*P* < 0.05).

**Table 3:** The effects of FA supplementation on growth performance of piglets

Items	Diquat –		Diquat +		SEM	*P*-value		
	Control	FA	Control	FA		FA	Diquat	Interaction
Days 1 to 14								
15 d BW, kg	21.38	22.03			0.34	0.35		
ADG, g/d	570.81^b^	631.70^a^			12.90	0.02		
ADFI, g/d	1,051.96	1,090.68			17.71	0.28		
F/G	1.82^a^	1.73^b^			0.02	0.02		
Days 15 to 19								
20 d BW, kg	24.49	24.75	19.40	21.67	0.52	0.09	< 0.01	0.11
ADG, g/d	701.50	632.00	−323.00	−146.29	90.42	0.47	< 0.01	0.10
ADFI, g/d	1,253.78	1,299.78	287.95	470.14	87.16	0.04	< 0.01	0.22

*BW* Body weight, *ADG* Average daily gain, *ADFI* Average daily feed intake, *F/G* Feed-gain ratio

^a,b^Mean values within a row with unlike superscript letters are significantly different (*P* < 0.05), *n* = 8

### Liver injury index

**Fig. 1:**
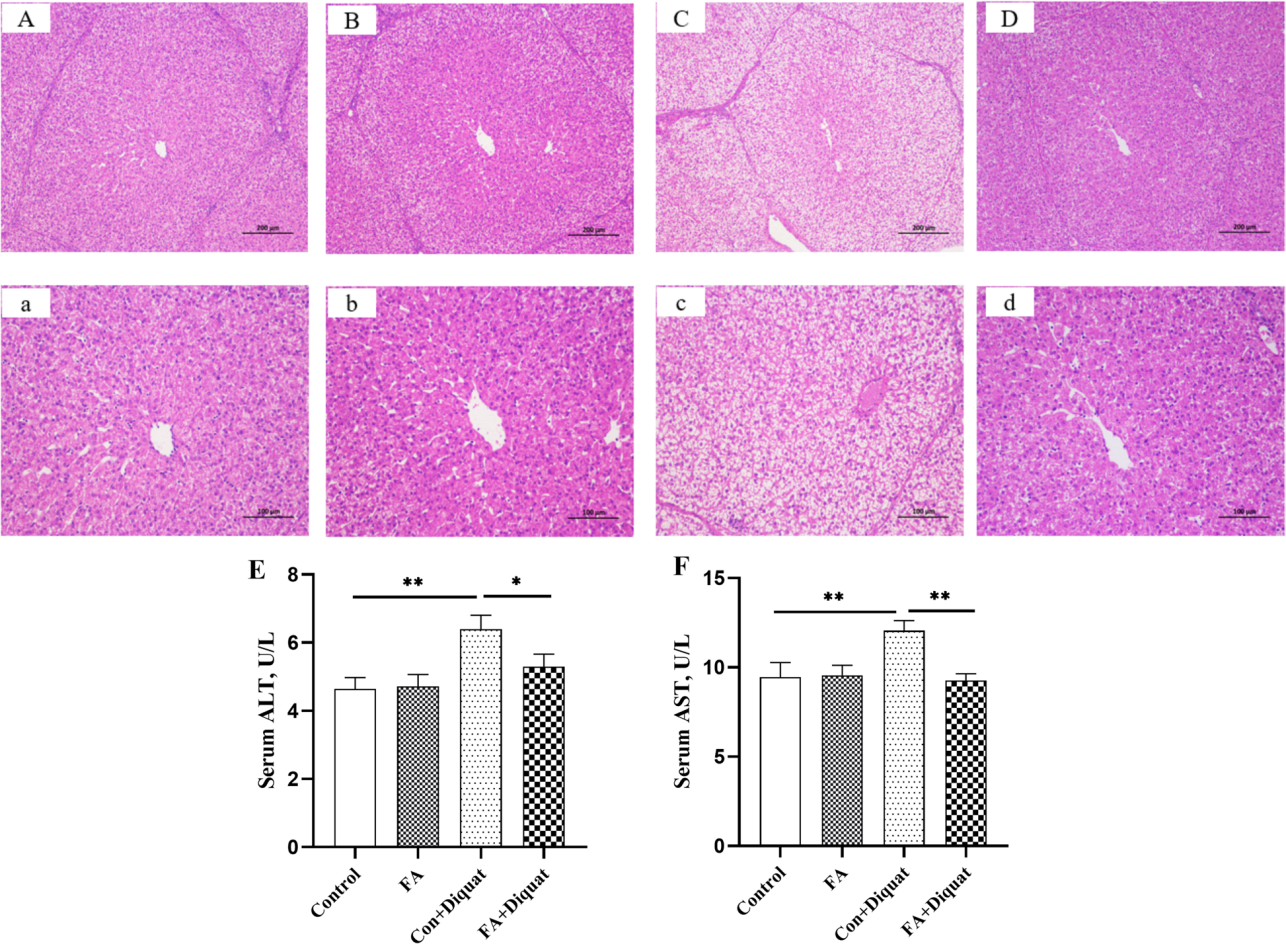
Effects of FA supplementation on hepatic injury in piglets.** A**–**D** 100 × ; **a**–**d** 200 × ; **A** and **a** Control group; **B** and **b** FA group; **C** and **c** Diquat-challenged group; **D** and **d** Diquat-challenged group supplemented with FA. ALT: alanine aminotransferase; AST: aspartate aminotransferase. Data are expressed as the means plus SEM (*n* = 8). ^*^P < 0.05, ^**^P < 0.01

To investigate the protective effects of FA on liver injury in diquat-challenged piglets, we measured hepatic morphology and transaminase activity. As shown in Fig. 1, hepatic tissues of piglets exhibited intact structure and dense cytoplasm in the control and FA groups (Fig. 1A and B). In contrast, the liver in the diquat group showed obvious balloon-like changes and severe cytoplasmic vacuolation (Fig. 1C), while the FA + diquat group showed no significant changes compared with the control group (Fig. 1D). Serum transaminase activity serves as a crucial indicator of liver damage [17]. Diquat injection significantly increased the activity of ALT and AST compared with control piglets (*P* < 0.05; Fig. 1E and F). Under diquat challenge condition, dietary FA supplementation mitigated the diquat-induced increase in ALT and AST activity (*P* < 0.05).

### Antioxidant indicators in serum and liver

**Fig. 2:**
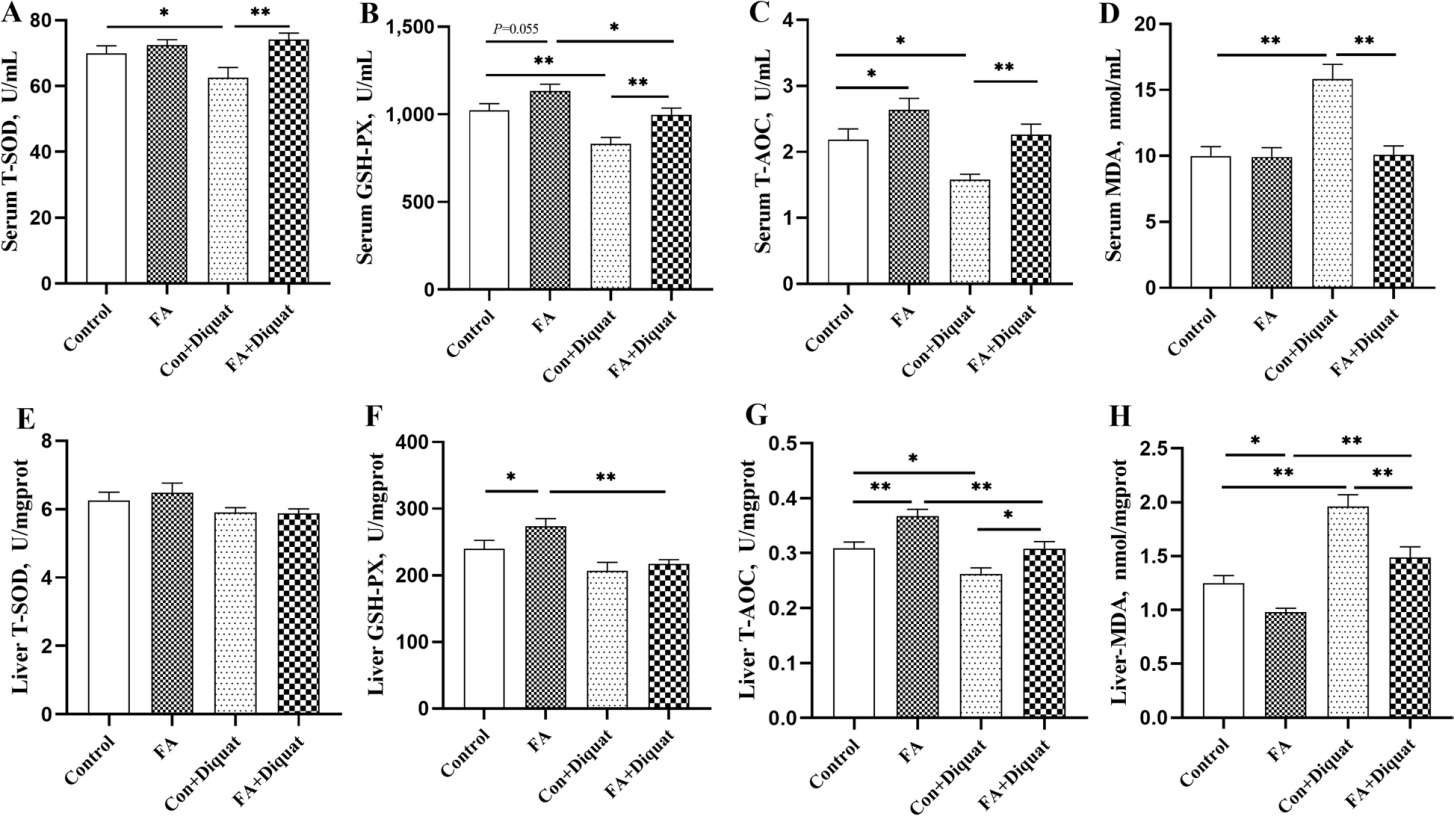
Effects of FA supplementation on antioxidant related indexes of piglets.** A**–**D** The levels of T-SOD, GSH-Px, T-AOC and MDA in serum. **E**–**H** The levels of T-SOD, GSH-Px, T-AOC and MDA in the liver. Data are expressed as the means plus SEM (*n* = 8). ^*^P < 0.05, ^**^P < 0.01

Serum concentrations of T-SOD, GSH-Px, T-AOC, and MDA are presented in Fig. 2A–D. Compared with the control group, activities of GSH-Px (*P* = 0.055) and T-AOC (*P* < 0.05) were elevated in FA group. Diquat lead to reduced activities of GSH-Px (*P* < 0.01), T-SOD (*P* < 0.05) and T-AOC (*P* < 0.05), while MDA concentration was markedly increased (*P* < 0.01). FA supplementation significantly increased GSH-Px, T-SOD and T-AOC activity (*P* < 0.01) and decreased MDA concentration (*P* < 0.01) in piglets exposed to diquat. In addition, the antioxidant capacity in the liver was assessed, which is depicted in Fig. 2E–H. GSH-Px activity (*P* < 0.05) and T-AOC capacity (*P* < 0.01) were increased, while MDA concentration (*P* < 0.05) was decreased in the liver of piglets supplemented with FA compared to the control group. Diquat-induced stress resulted in a notable reduction in T-AOC capacity (*P* < 0.05) and a significantly increase in MDA concentration (*P* < 0.01) in the liver. FA supplementation significantly increased T-AOC capacity (*P* < 0.05) and decreased MDA level (*P* < 0.01) in the liver of piglets exposed to diquat.

### mRNA expression of antioxidant-related genes in liver

To investigate the impact of feeding FA on Nrf2 signaling in piglet liver, we measured the expression levels of antioxidant-related gene. As shown in Table 4, FA and diquat have significant interaction on *Keap1* (*P* < 0.05) mRNA expression. Injection with diquat lead to increase in the relative expression of *Keap1* (*P* < 0.01), while FA supplementation reduced *Keap1* expression under diquat-stress condition (*P* < 0.05).

**Table 4:** Relative expression levels of antioxidant related genes involved in the liver of piglets

Items	Diquat –		Diquat +		SEM	*P*-value		
	Control	FA	Control	FA		FA	Diquat	Interaction
*Keap1*	1.00^b^	1.15^b^	1.62^a^	1.15^b^	0.08	0.237	0.027	0.025
*Nrf2*	1.00	1.35	0.69	0.97	0.07	0.019	0.013	0.796
*HO-1*	1.00	1.32	0.92	1.16	0.06	0.013	0.259	0.721
*NQO1*	1.00	1.35	0.82	0.94	0.08	0.123	0.059	0.457

*Keap1* Kelch-like ECH-associated protein 1; *Nrf2* NF-E2-related factor-2; *HO-1* Heme oxygenase 1; *NQO1* NAD[P]H-quinone oxidoreductase 1

^a,b^Mean values within a row with unlike superscript letters were significantly different (*P* < 0.05), *n* = 8

### Antioxidant-related protein expression in liver

**Fig. 3:**
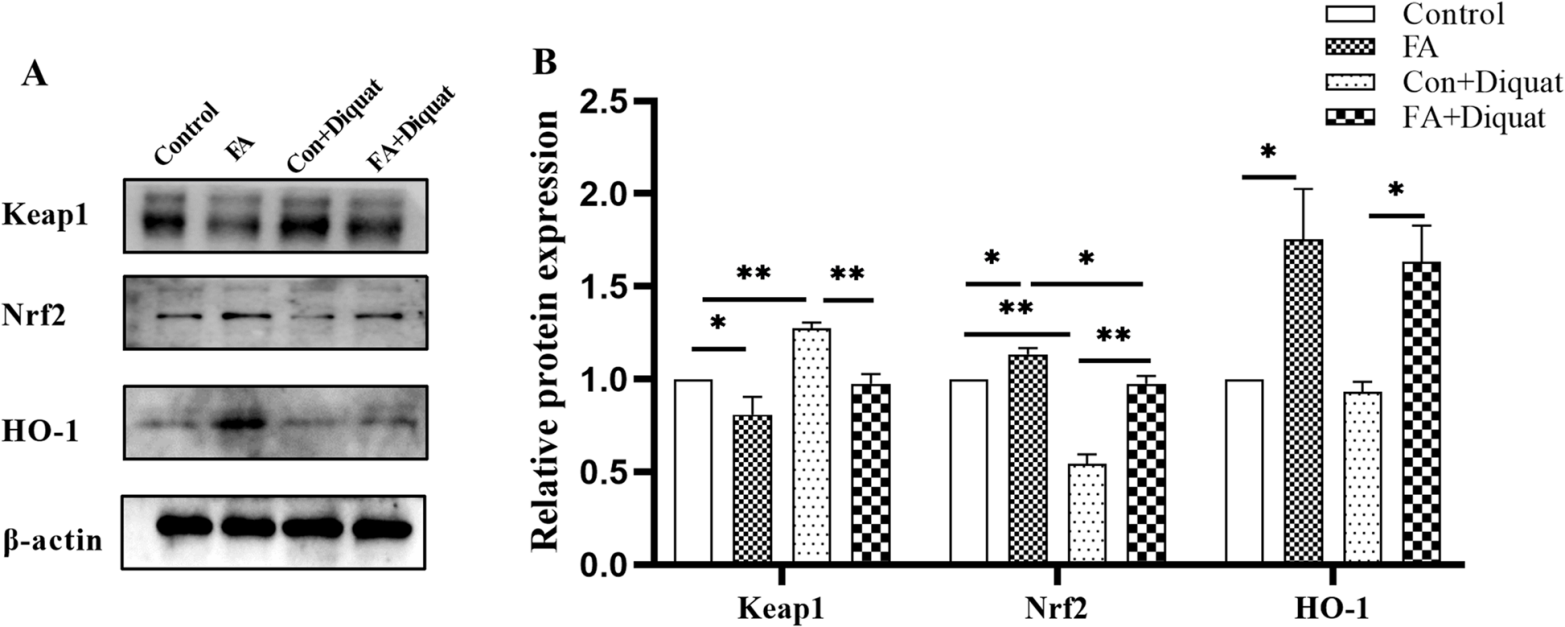
Relative expression levels of antioxidant-related proteins in the liver. **A** and **B** Protein abundance of Keap1, Nrf2, HO-1 and NQO1 in the liver. Data are expressed as the means plus SEM (*n* = 8). ^*^P < 0.05, ^**^P < 0.01

Western blotting results (Fig. 3) showed that FA significantly enhanced the relative protein expression levels of Nrf2 and HO-1, and reduced Keap1 protein expression (*P* < 0.05) compared with the control group. Diquat treatment significantly increased Keap1 protein expression (*P* < 0.01), and decreased Nrf2 protein expression (*P* < 0.01). However, FA supplementation mitigated the diquat-induced increase in Keap1 protein expression (*P* < 0.05) and increased the protein expression of Nrf2 and HO-1 (*P* < 0.05).

### ROS concentration and apoptosis proportion in liver cells

**Fig. 4:**
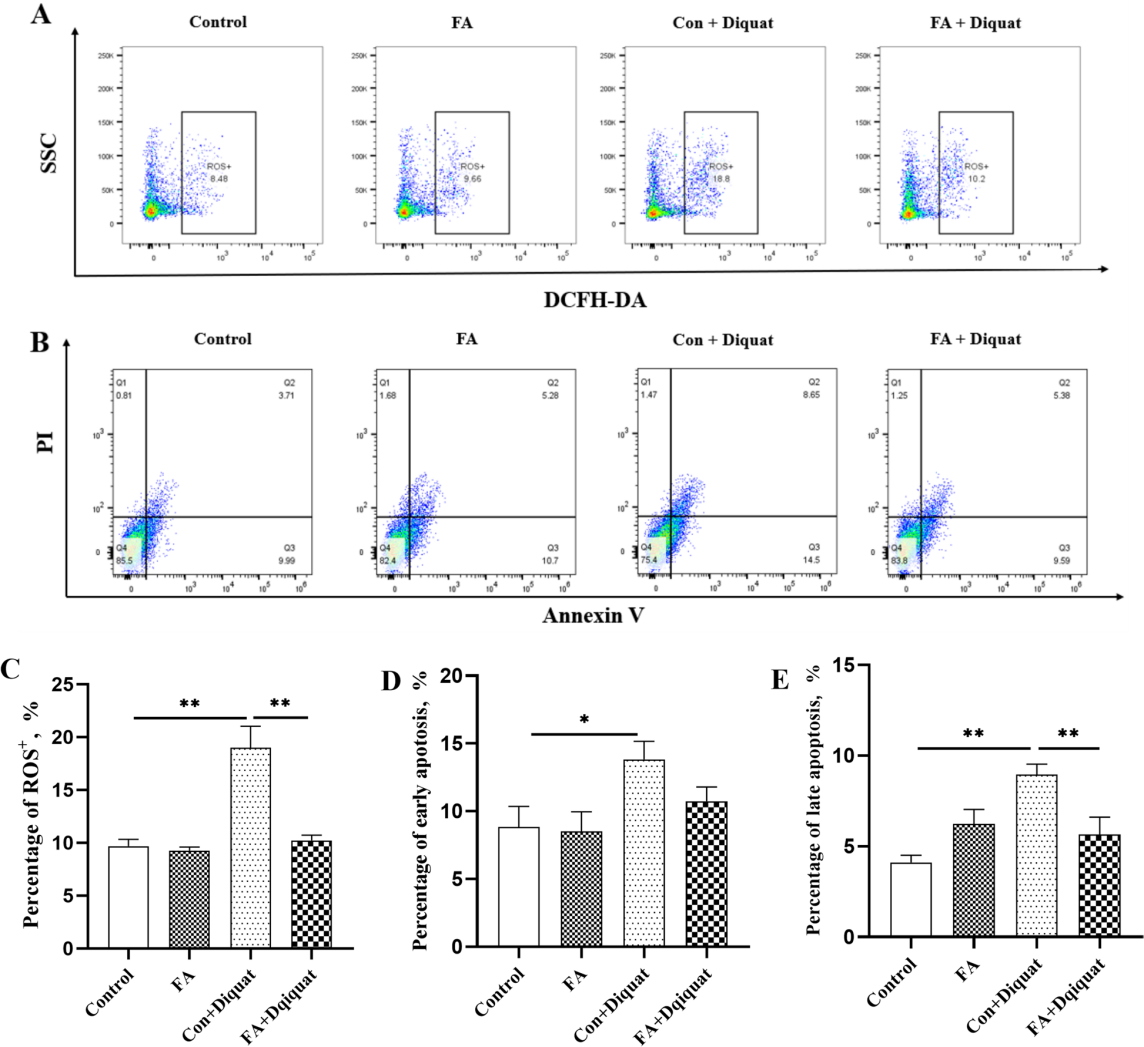
Effects of FA supplementation on the liver ROS level and apoptosis in the piglets.** A** and **C** Flow cytometry analysis of ROS in the liver; **B**, **D**, and **E** Flow cytometry analysis of apoptosis, proportion of apoptotic cells in early period (**D**) and in late period (**E**) in the liver. Data are expressed as the means plus SEM (*n* = 8). ^*^P < 0.05, ^**^P < 0.01

As shown in Fig. 4A and B, diquat treatment significantly increased ROS level in the liver (*P* < 0.01). Dietary FA supplementation significantly inhibited the diquat-induced increase in ROS level (*P* < 0.01). In addition, the proportion of apoptosis in the early (Q3, Fig. 4D) and late (Q2, Fig. 4E) period under diquat challenge were significantly higher than those in the control group (*P* < 0.05). Interestingly, FA supplementation significantly inhibited the increased hepatocytes apoptosis in diquat-injected pigs (*P* < 0.01).

### LDH and cytokine concentrations in serum

**Fig. 5:**
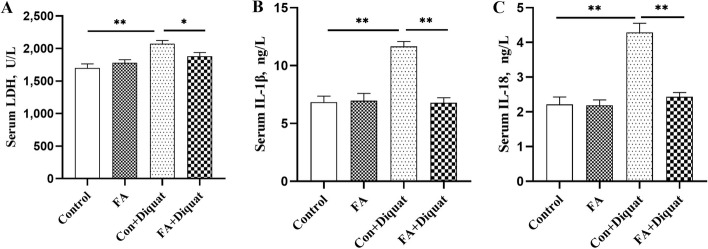
Effects of FA supplementation on serum LDH and cytokine concentrations in piglets. **A** The levels of LDH in serum; **B** and **C** The concentrations of IL-1β and IL-18 in serum. Data are expressed as the means plus SEM (*n* = 8). ^*^P < 0.05, ^**^P < 0.01

The data on LDH and cytokine concentrations of IL-1β and IL-18 are shown in Fig. 5. Compared with the control group, the diquat challenge resulted in a significant increase in LDH, IL-1β and IL-18 concentrations in serum (*P* < 0.01). However, FA significantly inhibited the increased concentrations in LDH, IL-1β and IL-18 under diquat challenge (*P* < 0.05).

### Expression of pyroptosis-related genes in liver

The relative mRNA levels of pyroptosis-related genes in the liver are shown in Table 5. FA and diquat have significant interaction on *Caspase-1 *mRNA expression (*P* < 0.01). Compared with the control group, diquat-induced oxidative stress significantly elevated the mRNA expression of *Caspase-1* in the liver (*P* < 0.05). FA supplementation significantly attenuated the diquat-induced increase in mRNA expression of *Caspase-1* (*P* < 0.05).

**Table 5:** Relative expression levels of pyroptosis related genes involved in the liver of piglets

Items	Diquat –		Diquat +		SEM	*P*-value		
	Control	FA	Control	FA		FA	Diquat	Interaction
*NLRP3*	1.00	0.83	1.46	0.79	0.10	0.033	0.267	0.183
*ASC*	1.00	1.04	1.35	1.10	0.13	0.713	0.472	0.612
*Caspase-1*	1.00^b^	1.08^b^	1.91^a^	0.72^b^	0.12	0.005	0.136	0.002
*GSDMD*	1.00	1.04	2.92	0.90	0.32	0.096	0.132	0.083
*IL-1β*	1.00	0.93	1.80	1.02	0.14	0.112	0.097	0.085
*IL-18*	1.00	0.73	1.46	0.66	0.09	0.001	0.186	0.069

*NLRP3* Nod-like receptor protein 3, *ASC* Apoptosis-associated blotch-like proteins, *Caspase-1* Cysteinyl aspartate specific proteinase-1, *GSDMD* Gasdermin-D, *IL-1β* Interleukin-1β, *IL-18* Interleukin-18

^a,b^Mean values within a row with unlike superscript letters were significantly different (*P* < 0.05), *n* = 8

## Discussion

Oxidative stress, deriving from early weaning, environmental changes, and exposure to toxic, exerts detrimental effects on piglet’s health, posing a serious threat to their performance and life safety [18, 19]. Finding prospective antioxidants has been crucial to foster the sustainable development of the pig industry. FA has been widespread applied as a functional additive in livestock and poultry farming [20–22]. Previous study demonstrated that dietary supplementation with FA increased ADG in heifers up to 21% [20]. In addition, 100 mg/kg FA enhanced the final body weight, ADG, and reduced the F/G of Tianfu broilers [22]. In this study, dietary supplementation with FA significantly improved growth performance of piglets, which is consistent with the results in previous study [19, 21, 22]. Furthermore, FA partially mitigated the adverse impact of diquat on growth performance of piglets.

The liver plays an important role in the growth and development of piglets, and oxidative stress impairs liver function [23]. In this study, we observed a significant increase in serum ALT and AST activity following diquat treatment, indicating increased permeability occurred in the hepatocyte membrane. Accordingly, Mahmoud et al.’s findings confirmed that FA inhibited methotrexate-induced increases in AST and ALT in rats [13]. In this experiment, FA not only improved the histological morphology of the liver under oxidative stress, but also effectively attenuated the diquat-induced elevation in the activity of ALT and AST. Thus, we infer that FA exerts a protective effect against oxidative damage in liver of piglets.

Oxidative stress is closely related to the onset and progression of liver diseases. Diquat was observed to stimulate ROS generation, disrupting the dynamic redox balance in the weaned piglets. Due to the accumulation of ROS, the liver is susceptible to oxidative damage [5, 24]. Consistent findings were observed in our study. Antioxidant enzymes, such as SOD and GSH-Px, play a pivotal role in reducing ROS level, thus mitigating oxidative damage. FA exhibits significant free radical scavenging ability [25]. In order to investigate the ameliorative effect of FA on cyclosporine-induced nephrotoxicity in rats, FA was found to decrease PC, MDA and NO levels in serum, and enhanced antioxidant enzyme activities in rat tissues [26]. Shu et al. found that FA mitigated LPS-induced pathological injury in hepatocytes through elevating levels of T-SOD, T-AOC and GSH [22]. Current study demonstrated that FA supplementation could boost the antioxidant capacity, thereby contributing to resistance against diquat-induced oxidative stress. This was evidenced by the ROS reduction, accompanied by increased activity of GSH-Px and T-SOD, and inhibition of MDA production. The findings coincide with the results reported by Wang et al. [16].

The Keap1/Nrf2 signaling pathway serves as a pivotal mechanism in cellular defense against oxidative stress. Oxidative stress triggers the activation of the Keap1/Nrf2 signaling pathway via stimulating the expression of crucial genes such as *HO-1* and *NQO1* [27, 28]. Studies have shown that FA can regulate the Keap1/Nrf2 signaling pathway and enhance activity of antioxidant enzymes, in order to protect host against oxidative stress triggered by ROS [16, 22]. In this study, FA promoted the expression of Keap1, Nrf2 and HO-1 in the liver of diquat-challenged piglets. Therefore, these results suggest that FA could enhance hepatic antioxidant capacity by stimulating downstream antioxidant enzymes through activation of the Keap1/Nrf2 signaling pathway.

Being a kind of cell death pathway, pyroptosis is predominantly mediated by the NOD-like receptor pyrin domain-containing 3 (NLRP3) inflammasome. Elevated ROS level activates the NLRP3 expression, and triggers cell death [29]. Cellular pyroptosis highly correlates with hepatic diseases, such as non-alcoholic steatohepatitis and liver fibrosis [30–32]. It has been demonstrated that sustained NLRP3 activation leads to poor growth and severe hepatic inflammation in mice. Furthermore, it has been confirmed that NLRP3 activation and NLRP3-dependent hepatocyte cellular pyroptosis represent novel mechanisms of liver injury and fibrosis [32]. While pyroptosis occurs, cellular membrane integrity is disrupted, resulting in the release of intracellular LDH and inflammatory factors [33, 34]. Hang et al. [35] found that oxidized LDL increased intracellular ROS, activated NLRP3 inflammasome, facilitated the maturation and secretion of IL-1β and IL-18, and increased LDH release in endothelial cells. Therefore, the increased proportion of late apoptosis suggests altered cell membrane permeability, indicative of potential pyroptosis or cell death. Considering the finding on LDH and cytokines (IL-1β and IL-18), we hypothesize that diquat may trigger hepatocyte pyroptosis in piglets. The data of hepatic pyroptosis paralleled the trend of ROS level, suggesting that diquat-induced ROS in the liver might infer the occurrence of pyroptosis. To further confirm cellular pyroptosis emerged in the liver, we used real-time PCR to assess pyroptosis-related gene expression. Our results indicate that oxidative stress up-regulated the gene expression of *Caspase-1*, indicating that diquat-induced hepatic oxidative stress is associated with pyroptosis. In addition, FA significantly attenuated diquat-induced hepatic cellular pyroptosis by down-regulating the expression of *Caspase-1* and inflammatory cytokines. These findings suggest that FA might inhibit hepatic pyroptosis by regulating the ROS/NLRP3 pathway.

We admit the duration of this study was too short to investigate growth performance of pigs. Instead, the current research mainly focuses on antioxidant capacity.

## Conclusions

In summary, current results demonstrate that FA can improve the antioxidant capabilities in piglets through modulation of related gene expression in the Keap1/Nrf2 signaling pathway (*Keap1*,* Nrf2 *and* HO-1*). Moreover, FA suppresses oxidative stress-induced hepatic cellular pyroptosis by diminishing gene expression (*NLRP3*, *GSDMD*, *Caspase-1*, *IL-1β*, and *IL-18*) within the ROS/NLRP3 pathway. Consequently, FA is anticipated to serve as a potent nutritional strategy for mitigating oxidative stress-induced liver damage.

## Data Availability

The data used to support the results of this study are available from the corresponding author according to reasonable requirements.

## Abbreviations

ADFI: Average daily feed intake
ADG: Average daily gain
ALT: Alanine aminotransferase
ASC: Apoptosis-associated blotch-like proteins
AST: Aspartate aminotransferase
BW: Body weight
Caspase-1: Cysteinyl aspartate specific proteinase-1
FA: Ferulic acid
GSDMD: Gasdermin-D
GSH-Px: Glutathione peroxidase
HO-1: Heme oxygenase 1
IL-1β: Interleukin-1β
IL-18: Interleukin-18
Keap1: Kelch-like ECH-associated protein 1
MDA: Malondialdehyde
NLRP3: Nod-like receptor protein 3
NQO1: NAD[P]H-quinone oxidoreductase 1
Nrf2: NF-E2-related factor-2
ROS: Reactive oxygen radicals
T-AOC: Total antioxidant capacity
T-SOD: Total superoxide dismutase
